# T and NK cells: two sides of tumor immunoevasion

**DOI:** 10.1186/1479-5876-11-30

**Published:** 2013-02-04

**Authors:** Doriana Fruci, Elisa Lo Monaco, Loredana Cifaldi, Franco Locatelli, Elisa Tremante, Maria Benevolo, Patrizio Giacomini

**Affiliations:** 1 Paediatric Haematology/Oncology Department, Bambino Gesù Children’s Hospital, IRCCS, Piazza Sant’Onofrio 4, Rome, 00165, Italy; 2 Laboratory of Immunology, Regina Elena National Cancer Institute, Via delle Messi d’oro 156, Rome, 00158, Italy; 3 Department of Paediatric Sciences, University of Pavia, Corso Strada Nuova 65, Pavia, 27100, Italy; 4 Laboratory of Pathology, Regina Elena National Cancer Institute, Via E. Chianesi 53, Rome, 00144, Italy

## Abstract

Natural Killer (NK) cells are known to reject several experimental murine tumors, but their antineoplastic activity in humans is not generally agreed upon, as exemplified by an interesting correspondence recently appeared in Cancer Research. In the present commentary, we join the discussion and bring to the attention of the readers of the Journal of Translational Medicine a set of recent, related reports. These studies demonstrate that effectors of the adaptive and innate immunity need to actively cooperate in order to reject tumors and, conversely, tumors protect themselves by dampening both T and NK cell responses. The recently reported ability of indoleamine 2,3-dioxygenase (IDO) and prostaglandin E2 (PGE2) expressed by melanoma cells to down-regulate activating NK receptors is yet another piece of evidence supporting combined and highly effective T/NK cell disabling. Major Histocompatibility Complex class I (MHC-I) molecules, including Human Leukocyte Antigen E (HLA-E), represent another class of shared activating/inhibitory ligands. Ongoing clinical trials with small molecules interfering with IDO and PGE2 may be exploiting an immune bonus to control cancer. Conversely, failure to simultaneously engage effectors of both the innate and the adaptive immunity may contribute to explain the limited clinical efficacy of T cell-only vaccination trials. Shared (T/NK cells) natural immunosuppressants and activating/inhibitory ligands expressed by tumor cells may provide mechanistic insight into impaired gathering and function of immune effectors at the tumor site.

## Commentary

A report published on March 15, and the following correspondence published October 11, 2012 in Cancer Research [1-3] revamp the old vexing question of Natural Killer (NK) cells and tumors. Do NK cells reject human tumors? Do they positively influence clinical outcome? Do tumors bother at all evading NK cells? In their original paper, Pietra and colleagues appear to answer yes to all these questions. They show that melanoma cells produce indoleamine 2,3-dioxygenase (IDO) and prostaglandin E2 (PGE2), two natural immunosuppressants that down-regulate activating NK receptors [1]. Whereas this is highly suggestive of an active immunoevasion strategy, in a subsequent letter to the Editor Sconocchia et al. emphasize the poor NK cell infiltration of most tumor lesions, including melanoma, a finding that is suggested to question the role of NK cells in contrasting solid tumor progression in humans [2]. In their conclusive authors’ reply, Pietra et al. attempt to reconcile these views. In extreme synthesis, they argue that NK cell disarmament and poor infiltration may be two sides of the same coin [3].

This interpretation is fully agreeable, but in our opinion it enlightens a particular case of a more general and widely inclusive concept. As thoroughly documented by Shanker and Marincola in a recent review, tumor rejection is a two-way, cooperative endeavor involving *both* innate and adaptive immunity: T and NK cells in the first place, but most certainly also other immune cells, including dendritic cells, macrophages, and neutrophils [4]. This is particularly evident in certain murine experimental tumor systems in which a direct communication axis has been identified between T and NK cells [5,6]. Somewhat reciprocal to this concept, we recently reviewed the available evidence that tumor immunoevasion requires the simultaneous derangement of both T and NK cells, as shown by the analysis of Major Histocompatibility Complex class I (MHC-I) phenotypes of human tumors [7]. Herein, we go on and argue that the function of a core set of shared mechanisms impairing T/NK cell functions begins to be unraveled. IDO, PGE2 and self MHC-I may be the prototypes of shared (T/NK) immunoevasion ligands. They may advance our understanding of how tumors are rejected or, alternatively, tolerated by the immune system on the whole, and not just T or NK cells separately considered.

**Figure 1:**
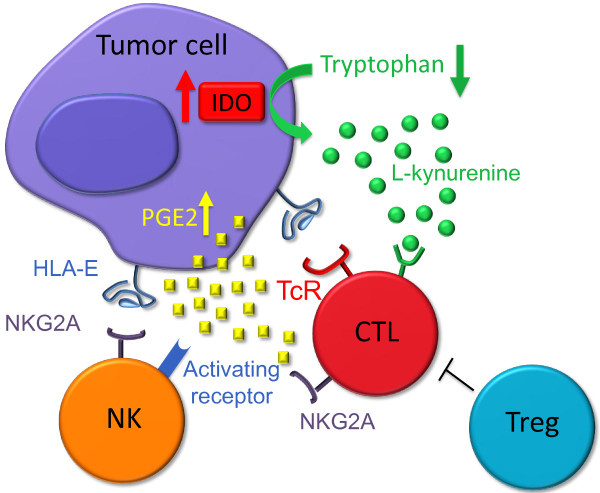
**IDO, PGE2 and HLA-E: a core set of immunosuppressants and immunomodulators acting on both T and NK cells.** Tumor cells create a potentially inhibitory microenvironment by producing IDO (causing tryptophan depletion and L-kynurenine accumulation), secreting PGE2, and expressing surface HLA-E. IDO and PGE2 inhibit T cell functions both directly and indirectly (through Treg cells), and down-regulate NK receptors, although with a different spectrum of activity. HLA-E binds the inhibitory receptor NKG2A, although it may also bind the activating receptor NKG2C. In addition, PGE2 is known to suppress IFN-γ production by T and NK cells, IL12/IL15 responsiveness of NK cells, and both production of, and responsiveness to, IL2 by T cells [1,8-10,15].

IDO and PGE2 have long been known to inhibit CD8^+^ T cells and increase suppressive, regulatory T cell (Treg) responses (reviewed in [8-11] and see Figure 1). Indeed, the combined inhibitory effect of IDO on T and NK cells had already been elucidated in 2002, in a seminal paper from the group led by the late GB Ferrara [12]. Expression of IDO and PGE2 is associated with progression and/or poor prognosis in many tumors including melanoma and colorectal carcinoma [8,9]. In both melanoma and colorectal carcinoma, IDO is detrimental to survival regardless of whether it is measured on cancer cells in primary lesions [13], or leukocytes (e.g. dendritic-like cells) in tumor-free draining lymph nodes ( [14] and reviewed therein).

**Figure 2:**
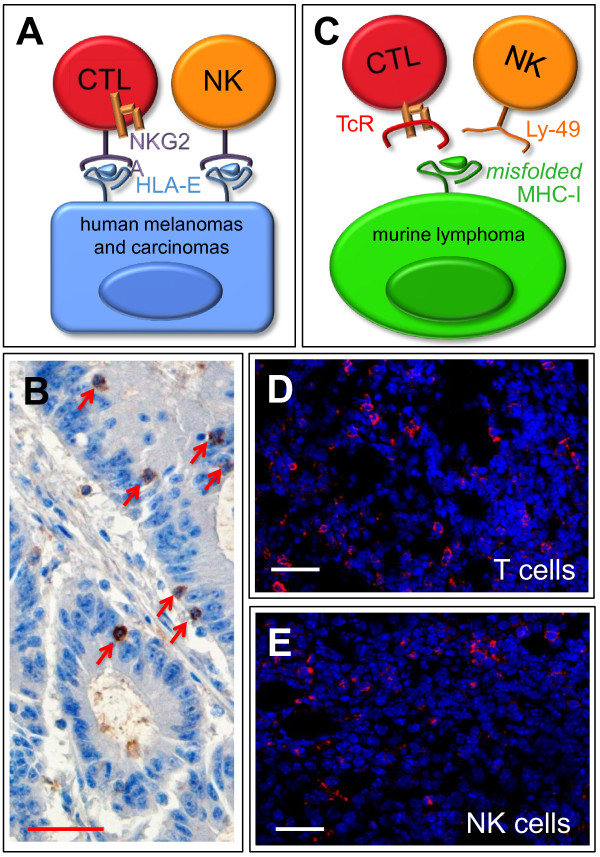
**Shared MHC-I ligands and tumor infiltration by T and NK cells.** Schematic diagrams depicting tumor recognition by (**A**) a single immune receptor (NKG2A) expressed by both T and NK cells, and (**C**) two distinct receptors: the TcR expressed by T cells and Ly-49 expressed by NK cells, both specific for a single class of immune ligands (MHC-I molecules). Immunohistochemical stain (**B**) of NKG2A^+^ cells infiltrating (arrows) a human colorectal carcinoma lesion. These are found in the context of CD8^+^ T cell infiltrates, whereas CD56^+^ cells were shown to be less than 2 per microscopic field, therefore marginally if at all contributing to NKG2A staining [16]. Counterstained with Hematoxylin. Fluorescent confocal microscopy imaging of CD8^+^ T cells (**D**) and NK1.1^+^ cells (**E**), in red, infiltrating RMA murine lymphoma tumors expressing misfolded MHC class I molecules. Counterstained with Hoechst 33342. Size bars: 40 μm in all panels.

Interestingly, the activating NK receptors inhibited by IDO and PGE2 are functionally counteracted by NKG2A, an inhibitory receptor utilized by both T and NK cells (Figure 1). Like IDO and PGE2, the NKG2A inhibitory ligand HLA-E is expressed and functional in tumor cells, including melanoma and colorectal carcinoma ( [15], reviewed therein, our own unpublished data, and see Figure 2A). HLA-E:NKG2A interactions are recapitulated *in vivo*: NKG2A^+^ T cells preferentially infiltrate a sub-group of HLA-E-high colorectal carcinomas (Figure 2B) with favorable 5-yr disease-free and overall survival [16].

These observations strongly suggest that different tumors preferentially tamper with shared T/NK cell immunosuppressants and ligands, e.g. the two sides of the immunoevasion coin are indeed poor tumor lysis and poor lymphoid infiltration, as argued by Pietra et al. and Sconocchia et al., but these immunoevasive strategies directly impact on *both* T and NK cells.

In the case of HLA-E:NKG2A, a favorable outcome of an inhibitory interaction may appear paradoxical, but this apparent paradox can be alleviated by considering, among other factors taken into account in the original publication [15], that NK cells cannot be simply switched on and off, but need rheostat-like tuning [17]. Unlicensed by default, they must undergo some kind of inhibitory receptor engagement prior to arming [18]. Thus, HLA-E expression, if too low, might itself promote tumor escape.

Whatever the instructive role of self HLA-E, one may predict that harnessing a selected group of critical immune checkpoints will simultaneously unleash a range of immune effectors against cancer cells. Indeed, interference with a single function (antigen trimming in the endoplasmic reticulum) of tumor cells induces a subtle conformational change in MHC-I ligands that activates both T and NK cells, leading to the concerted rejection of a murine transplantable lymphoma that is otherwise refractory to immune elimination [19]. Interestingly, the mechanism of action involves triggering and relief of inhibition, in T and NK cells respectively, by a single ligand (Figure 2C). This model is particularly relevant to the present issue, since tumor-rejecting T and NK cells are both highly lytic, and rapidly (within a few hours) convene at the tumor site ( [19]; Figure 2D and E).

Although common ligands and the T/NK cell crosstalk have been successfully exploited to reject murine tumors, a question remains unanswered: will similar strategies result in significant clinical benefit in humans? So far there are no startling reported results in clinical trials. T cell-oriented (peptide) vaccination has indeed failed to meet expectations [20]. One reason may be the intrinsic weakness of a T cell-only anti-tumor response. On the other hand, we are not aware of clinical trials deliberately aimed at directly triggering both T and NK cells. Online search ( http://www.clinicaltrials.gov/) reveals that at least 7 ongoing trials make use of IDO-specific inhibitors and vaccines. In many cases, results are due shortly (Table 1). As to PGE2, a major product of cyclooxygenase 2 (COX-2) activity [9], anti-COX treatments are expected to impact not only on cancer cell-autonomous events (e.g. promote/restore apoptosis on the one hand, and quench angiogenesis and aberrant growth signaling on the other), but also on the patient’s immune system. The scientific community is well aware that immune restoration may come as an important bonus of anti COX-2 clinical trials [9]. As of January 10, 2013, ongoing anti COX trials are 473 (http://www.clinicaltrials.gov/; search string: COX inhibitors AND cancer). Although online search does not readily identify anticipated endpoints for T/NK cell assessment, the results of IDO and PGE2 trials are eagerly awaited. More in general, finding additional and common T/NK cell immune checkpoints may pave the way for combination and/or low-dose therapeutic schedules, possibly resulting in additive or synergistic anti-tumor effects, and reduced toxicity.

**Table 1:** **Ongoing clinical trials with IDO inhibitors**^**1**^

**Trial number**	**Treatment**	**Tumor**	**status**	**Estimated completion**	**Sponsor/Institution**
NCT01219348	peptide^2^	NSCLC^3^	recruiting	June 2012	Herlev Hospital, Copenhagen, Denmark
NCT01685255	INCB024360^4^	Ovarian cancer	recruiting	Nov 2015	Incyte Corporation, Wilmington, DE
NCT01543464	peptide^2^	melanoma	recruiting	Sept 2014	Herlev Hospital, Copenhagen, Denmark
NCT00739609	1-methyl-D-tryptophan^5^	Various solid tumors	terminated	Oct 2012	Vanderbilt University, Nashville, TN and New Link Genetics Corporation, Ames, IA
NCT00567931	1-methyl-D-tryptophan^5^	Various solid tumors	recruiting	undefined	Lee Moffit Cancer Center, Tampa, FL and Virginia Commonwealth University, Richmond, VA.
NCT01604889	INCB024360^4^	melanoma	recruiting	Feb 2015	Various locations, Incyte Corporation, Wilmington, DE
NCT01195311	INCB024360^4^	Various solid tumors	Active, recruiting	Mar 2013	Incyte Corporation, Wilmington, DE

^1^ Last searched online (http://www.clinicaltrials.gov) on January 10, 2013. Only trials involving patient treatment were selected. No observational trials are included. Search string: “IDO”.

^2^ Vaccination scheme against an IDO epitope.

^3^ Non-Small Cell Lung Cancer.

^4^ Small molecule inhibiting IDO enzymatic activity.

^5^ A classical IDO inhibitor.

In conclusion, the quality of the immune infiltrate (including the *in situ* interplay of immune ligands and their receptors, particularly those shared by T and NK cells) is in our opinion far more important than the absolute NK cell count in the tumor. Appropriate immunotherapeutic tactics are needed to overcome at the same time poor gathering and poor killing. And this applies to effectors of both the innate and the adaptive immunity.

## Competing interests

The authors declare that they have no competing interests.

## Authors’ contribution

ELM, LC, ET and MB designed and performed the experiments and provided a critical reappraisal to the pertinent literature. DF, FL and PG drafted the manuscript. The text was edited and approved by all the authors.
